# Dietary patterns in India: a systematic review

**DOI:** 10.1017/S0007114516001598

**Published:** 2016-05-05

**Authors:** Rosemary Green, James Milner, Edward J. M. Joy, Sutapa Agrawal, Alan D. Dangour

**Affiliations:** 1 Department of Population Health, London School of Hygiene & Tropical Medicine, Keppel Street, London WC1E 7HT, UK; 2 Leverhulme Centre for Integrative Research on Agriculture and Health (LCIRAH), London WC1H 0PD, UK; 3 Department of Social and Environmental Health Research, London School of Hygiene & Tropical Medicine, 15-17 Tavistock Place, London WC1H 9SH, UK; 4 Public Health Foundation of India, Delhi NCR, Plot No. 47, Sector 44, Institutional Area Gurgaon 122002, India

**Keywords:** Diets, Dietary pattern analyses, India, Systematic reviews

## Abstract

Dietary patterns analysis is an emerging area of research. Identifying distinct patterns within a large dietary survey can give a more accurate representation of what people are eating. Furthermore, it allows researchers to analyse relationships between non-communicable diseases (NCD) and complete diets rather than individual food items or nutrients. However, few such studies have been conducted in developing countries including India, where the population has a high burden of diabetes and CVD. We undertook a systematic review of published and grey literature exploring dietary patterns and relationships with diet-related NCD in India. We identified eight studies, including eleven separate models of dietary patterns. Most dietary patterns were vegetarian with a predominance of fruit, vegetables and pulses, as well as cereals; dietary patterns based on high-fat, high-sugar foods and more meat were also identified. There was large variability between regions in dietary patterns, and there was some evidence of change in diets over time, although no evidence of different diets by sex or age was found. Consumers of high-fat dietary patterns were more likely to have greater BMI, and a dietary pattern high in sweets and snacks was associated with greater risk of diabetes compared with a traditional diet high in rice and pulses, but other relationships with NCD risk factors were less clear. This review shows that dietary pattern analyses can be highly valuable in assessing variability in national diets and diet–disease relationships. However, to date, most studies in India are limited by data and methodological shortcomings.

India has a rich and highly varied cuisine, and its various diets are strongly related to social identity, religion and other cultural factors^(^ ^1^ ^)^, as well as local agricultural practices and availability of diverse foods^(^ ^2^ ^)^. The ‘average diet’ in a country as large and geographically diverse as India is therefore likely to be of little relevance from a public health nutrition perspective. The identification of common dietary patterns relevant to population sub-groups in India, as well as their association with epidemiological profiles, is important.

A number of previous studies have used data from dietary surveys to identify distinct dietary patterns in India and to characterise the consumers of these patterns^(^ ^3^ ^)^. These studies have differed in both the data used to measure food consumption and the methods used to define dietary patterns. Previous studies have used a number of local and regional dietary surveys available in India that come from diverse sources and represent different sub-populations and time periods. Nationally representative data are rare because of the scale of undertaking national dietary surveys in such a large country.

In terms of methods, previous studies of dietary patterns have commonly used data-driven methods to identify foods that are typically consumed in combination with one another, or that tend to be consumed by the same type of individuals. However, there is no universally agreed method for such analyses, and several different statistical methods are in common use. These methods can be broadly divided into analyses of dominant ‘factors’ in the diet – for example principal component analysis (PCA) and factor analysis – or analyses of ‘clustering’ of foods in the diet – for example k-means clustering and latent class analysis (LCA)^(^ ^4^ ^)^.

This systematic review aims to draw together the existing literature of dietary modelling studies, to identify common dietary patterns reported for India and their primary socio-demographic characteristics. A greater knowledge of the main dietary patterns in India is important for nutrition and health policy makers to understand distributions and trends in diets within populations, as well as their relationships with health outcomes. This is especially important, as India undergoes a significant dietary transition from traditional diets to more ‘Western’ ways of eating and a concomitant epidemiological transition^(^ ^5^ ^)^. A secondary aim of this review is to explore whether Indian dietary patterns are associated with risk factors for nutrition-related non-communicable diseases (NCD).

## Methods

We performed a systematic review of published and grey literature to identify common dietary patterns in India and variations in these patterns by region, age and sex. We quantified relationships between membership of different dietary patterns and risk factors for NCD. The systematic review protocol can be accessed at http://sustaininghealth.lshtm.ac.uk/sahdi/.

### Search strategy

A number of search terms were used across multiple databases in order to identify studies that used modelling techniques to identify distinct dietary patterns in India. Search terms included combinations of ‘diet*’, ‘pattern*’ and ‘India*’ with specific terms including ‘food’, ‘intake’, ‘principal component*’, ‘pca’, ‘factor*’, ‘fa’, ‘cluster*’, ‘latent class*’ and ‘mixture model*’. Multi-database searching was carried out, across the EMBASE, EThoS, Global Health, IndMED, MEDLINE, PubMed, Scopus and ISS Web of Science databases. Other sources of literature were also searched, including Google Scholar, FAO, the World Bank and the International Food Policy Research Institute.

We included published peer-reviewed studies, grey literature such as dissertations, conference proceedings, reports and other non-peer-reviewed studies. No time cut-offs were imposed, so that articles from the beginning of the databases up to the date of search (10 July 2015) were eligible for inclusion. Papers were considered for inclusion if they were published in English, Hindi, Urdu or Punjabi and were original scientific research papers (review papers were not included). Included studies were required to use dietary survey data from India to estimate at least two distinct dietary patterns in humans. Studies of individual foods or nutrients rather than comprehensive dietary patterns and studies only of infant feeding were excluded. Reviews were hand-searched for other relevant references, as were the reference lists of included studies.

### Data extraction

A database was compiled containing all relevant information from the included studies. This database included the following fields: study authors, year, journal title or publication format, geographical area, years data were collected, specific populations studied, statistical methods, food groups used, number and description of the dietary patterns identified, characteristics of consumers of each dietary pattern (if available), whether the patterns were related to any health outcomes or risk factors and any other relevant information about the study.

The review team leader (R. G.) conducted the search using the databases listed above, and this search was 50 % replicated by another review team member (J. M.) in order to check for consistency. Two members of the review team (R. G. and J. M.) extracted the data in duplicate from the studies and entered them into the database. Cross-checking of data extraction was performed on all included studies in order to confirm that identical data were extracted.

### Data analysis

The data collected were expected to be highly heterogeneous in nature, so quantitative synthesis was unsuitable. The results of the data extraction were summarised using narrative synthesis and presented in tables. Study quality was assessed through a checklist of eight criteria including clear description of methods and study population (online review protocol).

## Results

**Fig. 1:**
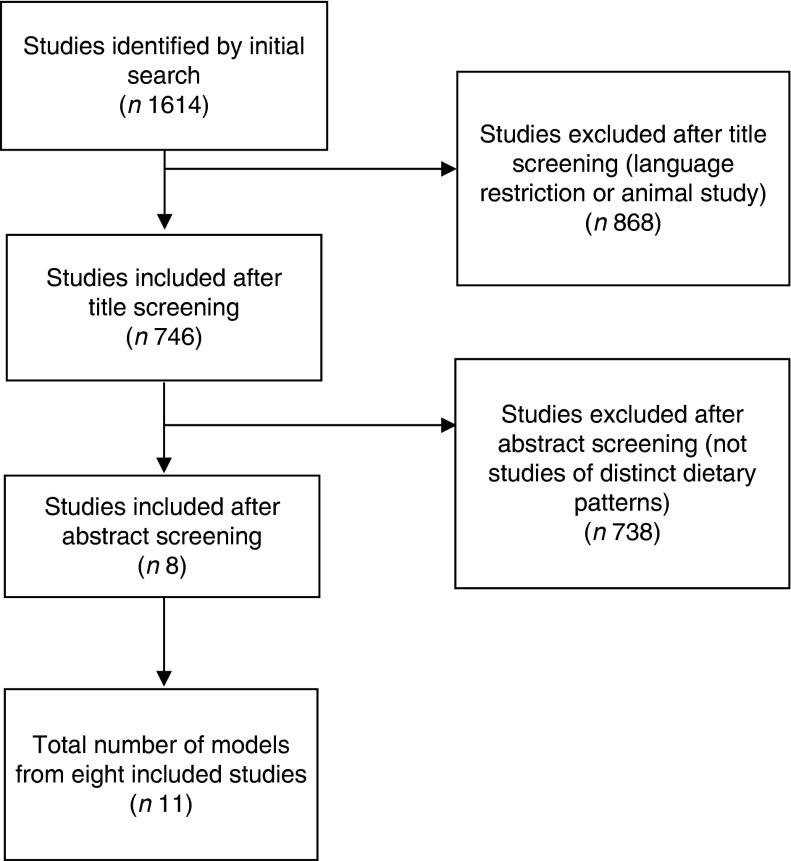
Studies included in the review.

The initial search identified 1614 references, of which 868 (54 %) were excluded as they were not published in the required languages or were not studies of diets in humans. The titles and abstracts of the remaining 746 identified studies were screened, with 738 being excluded because they did not model distinct dietary patterns. Eight studies that met all inclusion criteria were identified (Fig. 1). These eight studies contained a total of eleven separate models of dietary patterns in India: six studies provided one model each, one study provided three models for different geographical areas of India^(^ ^6^ ^)^ and one study provided separate models for men and women^(^ ^7^ ^)^. No additional papers were obtained by searching review articles or the reference lists of included papers, and all included studies were English language and from peer-reviewed journals rather than grey literature.

### Summary of included studies

The studies included eleven models of dietary patterns (Table 1). Models 6 and 9 focused on diets in children^(^ ^10^ ^,^ ^13^ ^)^, whereas the other models focused on adults^(^ ^6^ ^–^ ^9^ ^,^ ^11^ ^,^ ^12^ ^)^. Models 1 and 10 included males only^(^ ^7^ ^,^ ^8^ ^)^; models 5, 7 and 11 were of females only^(^ ^7^ ^,^ ^9^ ^)^; and models 2-4 and 8 were of males and females combined^(^ ^6^ ^,^ ^12^ ^)^. Only model 7 used data that were nationally representative, including data from all regions of India^(^ ^11^ ^)^. All regions of India were represented by at least three models, with the exception of the Northeast region for which data were only available from one model; thus, the Northeast was not analysed separately as a region. Models 1-5 and 8-11 used consumption data from the year 2000 onwards^(^ ^6^ ^–^ ^9^ ^,^ ^12^ ^,^ ^13^ ^)^, whereas models 6 and 7 used data from the 1990s^(^ ^10^ ^,^ ^11^ ^)^. Finally, models 1-6 and 8-11 used PCA as their method of defining dietary patterns^(^ ^6^ ^–^ ^10^ ^,^ ^12^ ^,^ ^13^ ^)^, whereas model 7 used LCA, a type of cluster analysis^(^ ^11^ ^)^.

**Table 1:** Summary of characteristics of included studies

Model no.	Sample size Demographic group	Region*	No. food groups	Method	No. patterns	Variance explained (%)	Study
1	284 Adult (M)	East	13	PCA	5	56	Chakraborty *et al*.^(^ ^8^ ^)^
2	824 Adult (M and F)	North	104	PCA	2	–	Daniel *et al*.^(^ ^6^ ^)^
3	743 Adult (M and F)	West	104	PCA	2	–	
4	2247 Adult (M and F)	South	104	PCA	2	–	
5	645 Adult (F)	East	24	PCA	3	27	Ganguli *et al*.^(^ ^9^ ^)^
6	538 Children	South	52	PCA	2	21	Kehoe *et al*.^(^ ^10^ ^)^
7	90 180 Adult (F)	North, South, East, West, Northeast	7	LCA	5	17	Padmadas *et al*.^(^ ^11^ ^)^
8	6581 Adult (M and F)	North, South, East, West	30	PCA	3	29	Satija *et al*.^(^ ^12^ ^)^
9	630 Children	West	12	PCA	5	55	Tupe & Chiplonkar^(^ ^13^ ^)^
10	2864 Adult (M)	East	13	PCA	6	59	Venkaiah *et al*.^(^ ^7^ ^)^
11	3525 Adult (F)	East	13	PCA	6	58	

M, male; F, female; PCA, principal component analysis; LCA, Latent class analysis; –, ‘variance explained’ was not reported.

* Region refers to geographical regions of India.

The number of food groups used by the researchers to define dietary patterns ranged from 7 to 104, with a mean number of forty-three food groups. The number of dietary patterns identified by each model ranged from two to six, with a mean of four dietary patterns per model. Not all models reported the proportion of the total variance in diets that was explained by the identified dietary patterns, but among the seven models that did report this the variance explained by all patterns ranged from 17 to 59 %, with a mean of 42 %.

Study quality was mixed, with only one study meeting all reporting criteria^(^ ^6^ ^)^ and two further studies meeting all but one^(^ ^11^ ^,^ ^13^ ^)^. Most studies provided adequate descriptions of models and statistical methods, and all studies met the basic requirements for reporting on study populations and geographical regions (although most were not nationally representative – see above). However, some studies failed to provide a detailed breakdown of the composition of each food group.

### Summary of dietary patterns

A total of forty-one dietary patterns were identified (Table 2). Most of the studies used comparable food groups to report the characteristics of the dietary patterns they identified, and these tended to be highly aggregated groups such as fruit, vegetables and dairy products. The dietary patterns identified were ranked 1–6 according to the proportion of the variance in diet they explained (Table 2). Pattern 1 explained 10–15 % of the variance in diets in the majority of studies. However, there was a great deal of variation in the food contents of the patterns identified, with some models characterised by traditional vegetarian food groups (models 2–4, 8 and 9), whereas others were characterised by meat and high-sugar/fat food groups (models 1, 5–7, 10 and 11).

**Table 2:** Summary of dietary patterns produced by included studies.

Model no.	Dietary pattern no.	Region*	Foods used to define pattern	Variance explained (%)
1	1	East	Snacks, sweets, fruit	14
	2		Fish, soft drinks	12
	3		Ghee, butter	11
	4		Fresh vegetables	10
	5		Dairy products	9
2	1	North	Fruit, dairy products, snacks	–
	2		Vegetables, pulses	–
3	1	West	Pulses, rice	–
	2		Sweets, snacks	–
4	1	South	Fruit, vegetables	–
	2		Snacks, meat	–
5	1	East	Vegetables, sweets, fruit, pulses, nuts, poultry, eggs	11
	2		Butter, oil, ghee	8
	3		Red meat, dairy products, cereals	8
6	1	South	Fruit, snacks, meat	9
	2		Rice, millet, dairy products	8
7	1	All	Meat, eggs, vegetables	9
	2		Pulses, eggs, leafy vegetables, dairy products	7
	3		Vegetables, meat	7
	4		Fruit, vegetables, dairy products, pulses	5
	5		Vegetables	5
8	1	All	Rice, nuts	–
	2		Other cereals, vegetables, fruit, dairy products, snacks, sweets	–
	3		Red meat, poultry, fish, eggs	–
9	1	West	Rice, pulses	–
	2		Snacks, fruit	–
	3		Wheat, dairy products, sprouts	–
	4		Millet, sprouts, leafy vegetables	–
	5		Sorghum, leafy vegetables	–
10	1	East	Dairy products, sugar	15
	2		Cereals, roots, vegetables, oils	10
	3		Cereals, pulses, nuts, seeds	9
	4		Cereals, leafy vegetables, fruit	8
	5		Fish, oils	8
	6		Meat, poultry	8
11	1	East	Dairy products, sugar	14
	2		Roots, vegetables	10
	3		Cereals, pulses, nuts, seeds	9
	4		Fish, oils	9
	5		Leafy vegetables, fruit	8
	6		Meat, poultry	8
Total	41			

–, ‘Variance explained’ was not reported.

* Region refers to geographical regions of India.

In total, twenty-nine dietary patterns were defined by predominantly vegetarian food groups, suggesting that vegetarian diets are still prevalent in India. These diets tended to be based on fruit, vegetables, pulses and cereals (mostly rice), with added dairy products, meat and eggs in many cases. The most common food groups across all patterns were vegetables (sixteen out of forty-one patterns), cereals (thirteen patterns) and fruit (ten patterns), meat (nine patterns), pulses (eight patterns) and dairy products (eight patterns). Less common were snacks and sweets, which were found in six patterns each.

### Differences between dietary patterns

We separated the models by region, year, age and sex in order to determine whether they showed a relationship with the distinguishing food groups found in Indian dietary patterns. The consumption of many food groups was variable across the studies from different regions. Sweets and snacks were more likely to characterise diets in the East and South, whereas fruit, vegetables, rice and pulses were more likely to characterise diets in the North and West. Dietary patterns from the East and South were also more likely to be defined by meat or fish consumption than those from the North and West.

We also divided the eleven models according to year of data, splitting the studies by whether they used data from before or after the year 2000 to provide a simplified method for exploring temporal trends given the limited number of studies. The patterns taken from more recent data were more likely to be characterised by consumption of wheat, fruit and sweets but less likely to be characterised by vegetables.

We found no difference in the most common food groups of dietary patterns according to sex or age: men and women had very similar dietary patterns, and there were not enough studies of children to make a valid comparison.

### Relationships with health outcomes

Six models reported associations between dietary patterns and nutrition/health outcomes (models 2–6 and 8; Table 3). The most common outcome studied (*n* 6) was body size (BMI, abdominal adiposity or waist circumference), and other studies reported associations with hypertension (*n* 2), diabetes (*n* 2) and cholesterol (*n* 1).

**Table 3:** Statistically significant relationships between dietary patterns and nutrition/health outcomes

Nutrition/health outcome	Model no.*	Dietary pattern no.	Foods used to define pattern	Direction of relationship
Body size (BMI, abdominal adiposity	2	1	Fruit, dairy products, snacks	+
or waist circumference)	3	2	Sweets, snacks	+
	4	2	Snacks, meat	+
	5	2	Butter, oil, ghee	+
	6	1	Fruit, snacks, meat	−
	8	3	Red meat, poultry, fish, eggs	+
Hypertension	2	1	Fruit, dairy products, snacks	+
	4	1	Fruit, vegetables	−
Diabetes (or pre-diabetes)	3	1	Pulses, rice	−
	3	2	Sweets, snacks	+
Cholesterol	5	1	Vegetables, sweets, fruit, pulses, nuts, poultry, eggs	−

* For details of model and dietary pattern numbers, see Tables 1 and 2.

Five of six models found that consumers of dietary patterns defined by high-fat and high-sugar foods such as sweets, snacks and animal products had significantly greater body size. The one remaining model found that a dietary pattern characterised by fruit, snacks and meat was associated with significantly smaller body size. Two models found a significant relationship between dietary patterns and hypertension, with one pattern characterised by vegetable and fruit consumption having an inverse relationship with hypertension, whereas a pattern defined by fruit, dairy products and snacks had a positive relationship with hypertension. A further two models found a significant relationship between dietary patterns and diabetes or pre-diabetes, with a pattern distinguished by rice and pulses associated with a lower risk of diabetes and a pattern characterised by sweets and snacks associated with a higher diabetes risk. Finally, a single model found that consumers of a varied dietary pattern defined by consumption of vegetables, sweets, fruit, pulses, nuts, poultry and eggs had significantly lower cholesterol.

## Discussion

This is the first systematic review of studies modelling dietary patterns in India. The results show that dietary pattern analysis is clearly warranted because of the wide variation in dietary patterns identified across the country. These range from more traditional vegetarian diets characterised by consumption of fruit, vegetables and pulses, to diets characterised by consumption of sweets, snacks and meat. The main differences in dietary patterns that we identified were due to the region of India (with diets in the North and West being more similar to one another, as were diets in the East and South). We found some differences in diets over time but very little difference between men and women.

These findings are consistent with what is already known about diets in India, namely that the majority of diets are vegetarian and high in fruit, vegetables and pulses^(^ ^14^ ^)^, with additional patterns being high in sweets and snacks and also containing meat. Our results also support existing evidence that there are large variations in dietary patterns between regions and that there is therefore little merit to attempting to define an average diet for the entirety of India. Diets also appear to be changing over time to provide more energy but also to contain a larger share of potentially less healthy foods such as those high in sugar^(^ ^5^ ^)^, although these results should be interpreted with caution, as only two models were available that used data from before 2000.

Our review of the associations between dietary patterns and health outcomes found several associations that are suggestive of dietary effects on health. The strongest evidence was of a relationship between dietary pattern and body size, but hypertension, diabetes and cholesterol levels were also found to be significantly related to particular dietary patterns. Common to many of the dietary patterns associated with less favourable risk factor profiles was the presence of snacks as a major dietary component. Snacks in India are usually high-fat, high-salt fried foods that may also be high in *trans*-fats^(^ ^14^ ^)^, and this may explain their relationship with a number of different health outcomes. On the other hand, a varied diet high in fruits, vegetables, pulses and nuts was associated with lower cholesterol, indicating that more traditional diets may have a healthier profile.

A major strength of this review is that a diverse range of studies was included in terms of populations. The review included studies of men, women and children from all regions of India, and with mostly recent sources of data. It was also able to combine data from a number of different modelling studies that had all used similar food groups to explore dietary patterns (and in some cases their association with nutrition/health outcomes). The review is therefore able to shed light on the diversity of dietary patterns that have been identified in India and some of their possible health impacts.

The validity of models used for analyses of dietary patterns has been proven in many cases^(^ ^3^ ^,^ ^15^ ^)^, and they have frequently been shown to have stronger relationships with health outcomes than individual foods or nutrients alone^(^ ^16^ ^,^ ^17^ ^)^. However, the models are dependent on the quality of the underlying food consumption data. In the present review, most studies relied on analyses of broad food group categories obtained through either FFQ (*n* 4), 24-h recall (*n* 2) or a combination of the two methods (*n* 2). Limitations of these methods include inherent measurement error (under-reporting or over-reporting of consumption) and potential subjectivity due to self-reporting. In particular, 24-h recall methods may not be representative of general dietary patterns over the longer term. The higher-quality studies tended to use more thorough methods of data collection, for instance the New Interactive Nutrition Assistant – Diet in India Study of Health method combining an interviewer-administered FFQ, open-ended questions, a food-preparer’s questionnaire and assistance with estimating portion sizes^(^ ^6^ ^)^.

In addition, there may be methodological limitations to defining valid dietary patterns^(^ ^18^ ^)^. In particular, PCA (which was used in all but one of the included studies) is a method that is highly useful for finding patterns in data, but it is not necessarily useful in terms of interpretation, because this method tends to separate foods into their factors of greatest importance, so that each pattern ends up being artificially composed of just a few distinguishing food items rather than representing an entire diet. Also, PCA cannot be used to allocate individuals to a given dietary pattern, and as such the mean levels of consumption of each food group in each pattern cannot be calculated^(^ ^4^ ^)^. It is possible to allocate individuals to patterns in PCA by rotating the factors orthogonally, but this was not done in all the included studies. Thus, the validity of the patterns produced is difficult to ascertain.

A possible improvement on this way of allocating individuals to dietary patterns would be to use a clustering method such as LCA (used by only one of the included models in this review). This can make the results easier to interpret, as the average consumption of each food group can be calculated for all dietary patterns, giving a more ready interpretation of the entire diet^(^ ^4^ ^)^. We would therefore recommend that future studies investigating dietary patterns consider using these methods as opposed to PCA for ease of interpretation, although these methods present their own challenges such as how to choose and label the most appropriate number of patterns, as this must be prespecified by the researcher.

The data available on nutrition and health outcomes and their links with dietary patterns were also limited, and we were therefore not able to draw firm conclusions about the relationships between Indian dietary patterns and health. However, the evidence on body size appears to point to the fact that emerging dietary patterns that include greater consumption of high-fat and high-sugar foods are linked with increasing obesity in particular, and potentially also hypertension and diabetes, whereas a more traditional diet, based on fruits, vegetables and pulses, may be linked to more positive NCD-related health outcomes. Future studies of Indian dietary patterns should seek to expand on these potential health impacts using high-quality dietary data and larger, more representative populations.

Finally, it is difficult to synthesise the results of models that have produced such divergent results. We were unable to find many commonalities between the patterns identified by the different studies, and this is most likely because of the huge variety in the numbers of food groups included and the data sources. Quantitative analysis was therefore impossible in this study. A more satisfactory option for future work would be to obtain the raw data from each study and pool these to create a single model of dietary patterns for all of India.

### Conclusion

This review has shown that dietary patterns in India are highly diverse, including traditional vegetarian patterns, those that incorporate high-fat, high-sugar foods and also meat. We also found large regional variations and some evidence of changes in patterns over time. The evidence of association between dietary patterns and nutrition or health impacts was sparse, but it did appear to indicate a relationship between patterns characterised by sweets, savoury snacks and meat and obesity, as well as potentially other CVD risk factors. However, the major limitations of data and methodology limit the conclusions that can be drawn from this work.

Future work would benefit from using larger and more representative food consumption data sets or to pool data from a number of sources in order to study the range of Indian dietary patterns in more detail, but in the meantime this review provides evidence that important variability between diets within a country can be captured using dietary patterns studies, and that such studies can help identify important links between diet and disease. This will be useful for health and nutrition policymakers in determining how to target dietary interventions to reduce disease burdens.

## References

[1] Vecchio MG, Paramesh EC, Paramesh H, et al (2014) Types of food and nutrient intake in India: a literature review. Indian J Pediatr 81, Suppl. 1, 17–22. 24928105 10.1007/s12098-014-1465-9

[2] Subramanyam MA, Kawachi I, Berkman LF, et al (2010) Socioeconomic inequalities in childhood undernutrition in India: analysing trends between 1992 and 2005. PLoS ONE 5, e11392. 20617192 10.1371/journal.pone.0011392 PMC2894973

[3] Moeller SJ, Reedy J, Millen AE, et al (2006) Dietary patterns: challenges and opportunities in dietary patterns research. J Am Diet Assoc 107, 1233–1239. 17604756 10.1016/j.jada.2007.03.014

[4] Newby PK & Tucker KL (2004) Empirically derived eating patterns using factor or cluster analysis: a review. Nutr Rev 62, 177–203. 15212319 10.1301/nr.2004.may.177-203

[5] Popkin BM, Horton S, Kim S, et al (2001) Trends in diet, nutritional status, and diet-related non-communicable diseases in China and India: the economic cost of the nutrition transition. Nutr Rev 59, 379–390. 11766908 10.1111/j.1753-4887.2001.tb06967.x

[6] Daniel CR, Prabhakaran D, Kapur K, et al (2011) A cross-sectional investigation of regional patterns of diet and cardio-metabolic risk in India. Nutr J 10, 12. 21276235 10.1186/1475-2891-10-12 PMC3042918

[7] Venkaiah K, Brahmam GN & Vijayaraghavan K (2011) Application of factor analysis to identify dietary patterns and use of factor scores to study their relationship with nutritional status of adult rural populations. J Health Popul Nutr 29, 327–338. 21957671 10.3329/jhpn.v29i4.8448 PMC3190363

[8] Chakraborty R, Bose K & Ulijaszek SJ (2009) Income level and food intake patterns among male Bengalee slum dwellers in Kolkata, India. Malays J Nutr 15, 19–25. 22691801

[9] Ganguli D, Das N, Saha I, et al (2011) Major dietary patterns and their associations with cardiovascular risk factors among women in West Bengal, India. Br J Nutr 105, 1520–1529. 21272403 10.1017/S0007114510005131

[10] Kehoe SH, Krishnaveni GV, Veena SR, et al (2014) Diet patterns are associated with demographic factors and nutritional status in South Indian children. Matern Child Nutr 10, 145–158. 23819872 10.1111/mcn.12046 PMC3920637

[11] Padmadas SS, Dias JG & Willekens FJ (2006) Disentangling women’s responses on complex dietary intake patterns from an Indian cross-sectional survey: a latent class analysis. Public Health Nutr 9, 204–211. 16571174 10.1079/phn2005842

[12] Satija A, Hu FB, Bowen L, et al (2015) Dietary patterns in India and their association with obesity and central obesity. Public Health Nutr 18, 3031–3041. 25697609 10.1017/S1368980015000312 PMC4831640

[13] Tupe R & Chiplonkar SA (2010) Diet patterns of lactovegetarian adolescent girls: need for devising recipes with high zinc bioavailability. Nutrition 26, 390–398. 19628369 10.1016/j.nut.2009.05.018

[14] Misra A, Singhal N, Sivakumar B, et al (2011) Nutrition transition in India: secular trends in dietary intake and their relationship to diet-related non-communicable diseases. J Diabetes 3, 278–292. 21649865 10.1111/j.1753-0407.2011.00139.x

[15] Hu FB (2002) Dietary pattern analysis: a new direction in nutritional epidemiology. Curr Opin Lipidol 13, 3–9. 11790957 10.1097/00041433-200202000-00002

[16] Newby PK, Muller D & Tucker KL (2004) Associations of empirically derived eating patterns with plasma lipid biomarkers: a comparison of factor and cluster analysis methods. Am J Clin Nutr 80, 759–767. 15321819 10.1093/ajcn/80.3.759

[17] Wirfalt E, Mattisson I, Gullberg B, et al (2000) Food patterns defined by cluster analysis and their utility as dietary exposure variables: a report from the Malmo Diet and Cancer Study. Public Health Nutr 3, 159–173. 10948383 10.1017/s1368980000000197

[18] Patterson BS, Dayton CM & Graubard BI (2002) Latent class analysis of complex sample survey data: application to dietary data. J Am Stat Assoc 97, 721–731.

